# CFD-aided design and hydrodynamic characterization of a single-use perfusion bioreactor for high density cell culture

**DOI:** 10.1186/s40643-026-01077-6

**Published:** 2026-06-03

**Authors:** Yongqiang Liu, Qingfeng Gu, Yu Liu, Guoqian Xu, Yingping Zhuang, Meijin Guo, Chao Li

**Affiliations:** 1 State Key Laboratory of Bioreactor Engineering, East China University of Science and Technology, 130 Meilong Rd., P.O. box 329#, Shanghai, 200237 People’s Republic of China; 2 School of Biotechnology, East China University of Science and Technology, 130 Meilong Rd., Shanghai, 200237 People’s Republic of China; 3 Alit Biotech (Shanghai) Co., Ltd, Building A8, 66 Yunkai Road, Songjiang District, Shanghai, 201600 People’s Republic of China

**Keywords:** Single-use bioreactor, Computational fluid dynamics, Culture of animal cells, High density culture, Shear strain rate, Perfusion culture

## Abstract

To address the challenges of high-density animal cell culture, this study developed a high-density culture system comprising a single-use bioreactor (SUB) with a nominal volume of 500 mL and a pulsed tangential flow filtration (ITF) unit. The reactor can be configured with two layers of 35 mm diameter impellers—either double Elephant Ear (EE-EE) or Elephant Ear combined with Ribbon (EE-RB). The flow field characteristics were rigorously characterized through CFD simulations (60–240 rpm; 90–480 mL) validated by experimental data. Engineering analysis revealed the system’s robust culture environment: ${k}_{L}a$ values ranging from 2 to 15 h^−1^ (60–180 rpm; 200–400 mL; 30–150 mL/min aeration), $P/V$ values from 0.1 to 10 W/m^3^, and mixing times between 1 and 10 s. Crucially, the system maintains a mild shear environment with an average shear strain rate (SSR) below 25 s^−1^, fully within the physiological tolerance range for mammalian cells. This low-shear, high-mass-transfer design was validated through perfusion cultures of CHO and HEK293 cells. The system achieved exceptionally high cell densities of 8.32 × 10^7^ cells/mL and 1.17 × 10^8^ cells/mL, respectively, with cell viability consistently exceeding 90%, demonstrating its suitability for high-intensity biopharmaceutical production.

**Graphical abstract:**

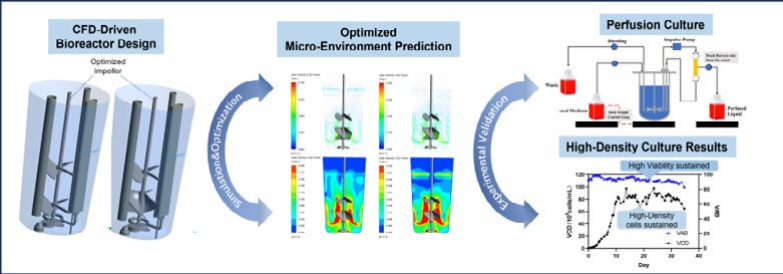

**Supplementary Information:**

The online version contains supplementary material available at 10.1186/s40643-026-01077-6.

## Introduction

Single-Use bioreactors are extensively utilized in the commercial production of monoclonal antibodies, viral vaccines, and recombinant protein therapeutics. Their widespread adoption is primarily attributed to their inherent operational flexibility, their capacity to significantly accelerate processing timelines, and their structural efficacy in eliminating cross-contamination risks (Eibl et al. 2010). For mammalian cell-based bioprocesses, the engineering metrics of a bioreactor—specifically mass transfer efficiency, shear stress distribution, global mixing, and power input—are pivotal determinants of productivity and cell viability (Junne and Neubauer 2018).

Adequate mass transfer is critical for regulating the exchange between gas and liquid phases, ensuring a sufficient dissolved oxygen supply while stripping inhibitory carbon dioxide to sustain cellular respiration. Concurrently, rapid mixing is essential to eliminate spatial gradients, ensuring the homogenous dispersion of nutrients and cells (Bach et al. 2017). Traditionally, enhancing mass transfer and mixing dictates an increase in agitation intensity. However, this intrinsically elevates the power input and generates intense localized shear forces. Mammalian cells, lacking a protective cell wall, are acutely sensitive to such hydrodynamic stresses. In high-shear environments, hydrodynamic forces translate into mechanical deformation stress on the cell membrane, triggering mechanotransduction pathways that alter morphology and metabolism, and can culminate in apoptosis or catastrophic membrane rupture (Vickroy et al. 2007; Strobl et al. 2021). Keane et al. (2003) found that when shear stress in the culture environment increased from 0.005 N/m^2^ to 0.8 N/m^2^, high Shear Strain Rate (SSR) negatively impacted CHO cell metabolism, resulting in a 51% decrease in recombinant protein yield, a 23.5% increase in glucose consumption rate, and a 45.6% increase in lactate production. Zhan et al. (2020) investigated the effect of SSR on erythropoietin production in HEK293 cells. The authors found that in low-shear environments, gene expression related to transcription and protein phosphorylation associated with product synthesis increased, leading to increased erythropoietin production. In high-shear environments, however, cells induced apoptosis through three pathways: endoplasmic reticulum stress, cytoskeleton reorganization, and extrinsic signaling pathways. The differentiation of mesenchymal stem cells (MSCs) is influenced by shear stress, with MSCs differentiating into osteoblasts under shear devices when shear stress increased from 0 to 2 dyn/cm^2^ within 2 min, with a total duration of 20 min. Conversely, when MSCs were exposed to shear stress increasing from 0 dyn/cm^2^ to 2 dyn/cm^2^ at the beginning of their culture, they differentiated into chondroblast cells, also over a 20-minute period (Lu et al. 2016; Yue et al. 2019). Accurate comprehension of engineering parameters related to the flow field characteristics of bioreactors is crucial for the structural design of cell bioreactors, optimization of culture processes, and enhancement of production scalability (Zhang et al. 2022).

Although certain parameters of bioreactors can be measured experimentally, accurate acquisition of parameters such as Shear Strain Rate (SSR) and Turbulence Eddy Dissipation (TED), which exhibit non-uniform distribution in space and time, often exceeds the scope of conventional experimental methods. Computational Fluid Dynamics (CFD) serves as an effective tool for assessing bioreactor performance, providing both global and local flow field data as well as critical engineering parameters. This method has seen rapid development in recent years and is widely applied in the design of bioreactors, performance validation, and bioprocess scale-up (Amer et al. 2019).

The cell perfusion system is capable of continuously renewing the culture medium and timely removing metabolic wastes, thus providing a stable and comfortable environment for cell growth and production in the bioreactor, enabling high-density culture of animal cells and efficient product expression. Studies indicate that in continuous perfusion culture, the viable cell density (VCD) of CHO cells is 1.6 times higher than that in batch or fed-batch cultures, with a 450% increase in product expression (Cha et al. 2021). Although perfusion culture has its drawbacks, such as longer culture duration and lower medium utilization efficiency, the cost of culture medium per unit product is nearly identical to that of fed-batch cultures (Xu et al. 2017b). Currently, Tangential Flow Filtration (TFF) and Alternating Tangential Flow Filtration (ATF) are the two predominant cell retention technologies in use (Bielser et al. 2018). Karst et al. (2016) found that TFF can achieve a monoclonal antibody concentration as high as 50% in the filtrate, compared to only 10% with ATF under the same conditions. However, the higher shear forces associated with TFF limit its application in cell and product separation, particularly in processes sensitive to shear forces, such as cell culture and product manufacturing. In TFF systems, peristaltic pumps are commonly used to generate higher shear forces, whereas ATF systems rely on creating chambers to provide the necessary driving force, leading to certain limitations in perfusion speed. Research has shown that by employing low-shear centrifugal pumps instead of peristaltic pumps to drive TFF systems, the screening limitations associated with higher shear forces can be effectively reduced to a level comparable to that of ATF systems (Wang et al. 2017).

To bridge this critical technological gap and resolve the longstanding trade-off between efficient mass transfer and shear-induced cellular damage, this study developed a highly novel animal cell perfusion culture system named Biogenstar. The core novelty of this system lies in its unique integration of a Single-Use bioreactor (SUB) with an innovative Single-Use Impulse Tangential-Flow Filtration (ITF) unit. Unlike conventional setups, this design specifically mitigates mechanical stress while sustaining high-intensity bioprocessing. Furthermore, this study provides a comprehensive and novel methodological framework by coupling computational fluid dynamics (CFD) with empirical validations. The stirred system within the bioreactor was systematically optimized to map and quantitatively characterize critical micro-environmental parameters related to mass transfer, shear, and mixing under various liquid volumes, stirring speeds, and aeration rates. Based on these unique hydrodynamic insights, highly efficient perfusion culture processes for CHO cells and HEK293 cells were developed and validated, systematically demonstrating the system’s superior capability to maintain high cell viability in a ultra-high-density environment.

## Materials and methods

### Biogenstar composition

#### Single-use bioreactor

**Fig. 1:**
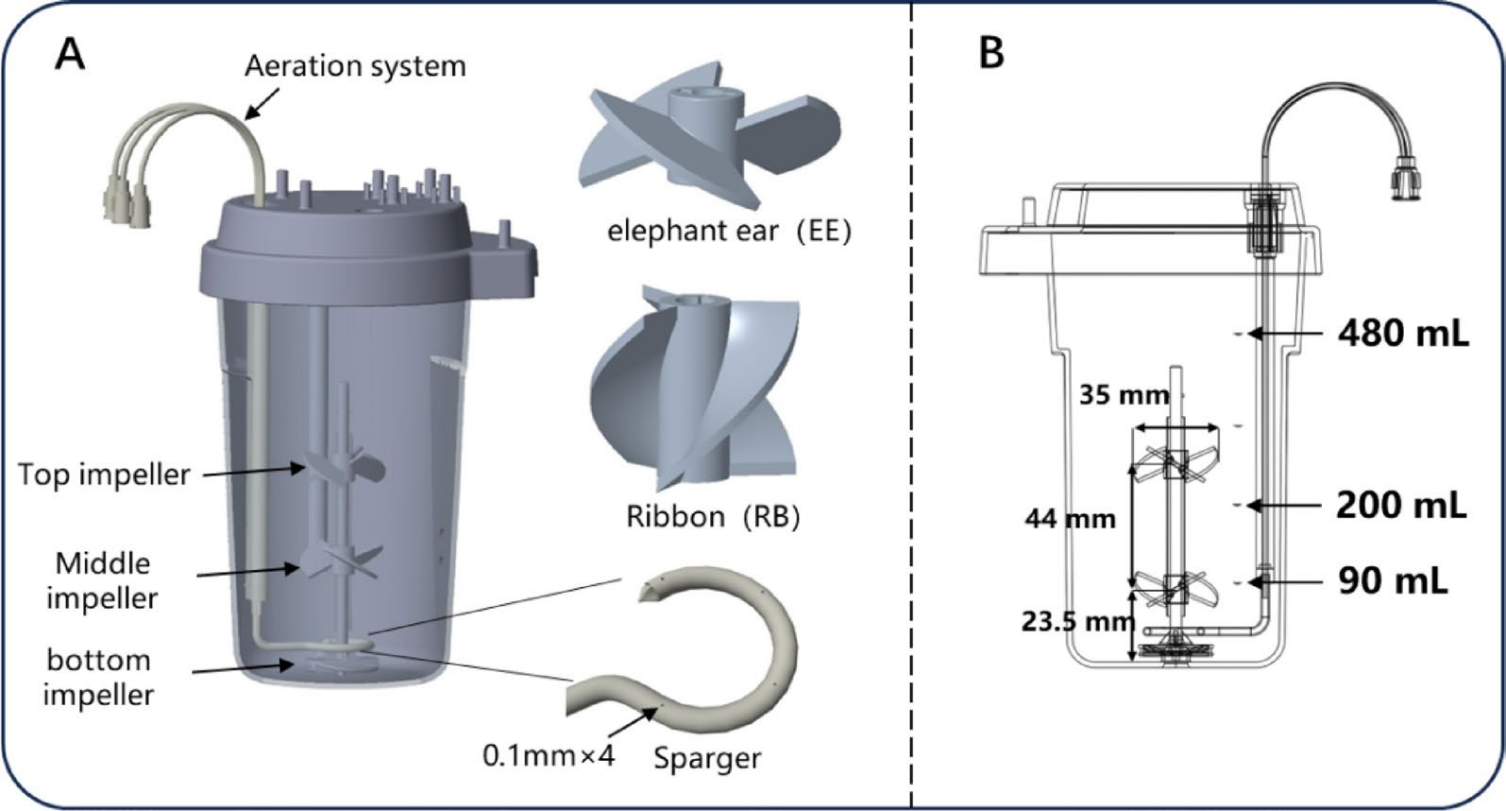
Structure of Single-Use Bioreactor (**A** is the 3D diagram,** B** is the 2D diagram)

The nominal volume of the single-use bioreactor (SUB) is 500 mL. Figure 1 shows the structure of the SUB, including the detailed dimensions of the impellers, liquid volume height, and the positioning of the impellers (top and bottom). The bioreactor is equipped with modular tubing and non-contact optical pH and dissolved oxygen (DO) electrodes. Inside the reactor, two layers of elephant ear impellers are installed, with a spacing of 44 mm between the two layers and a diameter of 35 mm for each impeller. The agitation system is driven by a magnetic rotor at the bottom, providing adequate gas transfer and mixing. Aeration is critical for high-density perfusion culture, which is provided through a gas manifold combining three gas tubes, one of which is equipped with an O-ring sparger featuring four 0.1 mm diameter holes, supplemented by overlay aeration in the headspace. The reactor vessel and the stirring impellers are made of polycarbonate (PC) material. All materials are sterilized using irradiation and then packaged aseptically. In addition, in this study, besides the elephant ear-elephant ear (EE-EE) impeller combination, an elephant ear-ribbon (EE-RB) configuration was also introduced as a control.

#### Impulse tangential-flow filtration perfusion system

**Fig. 2:**
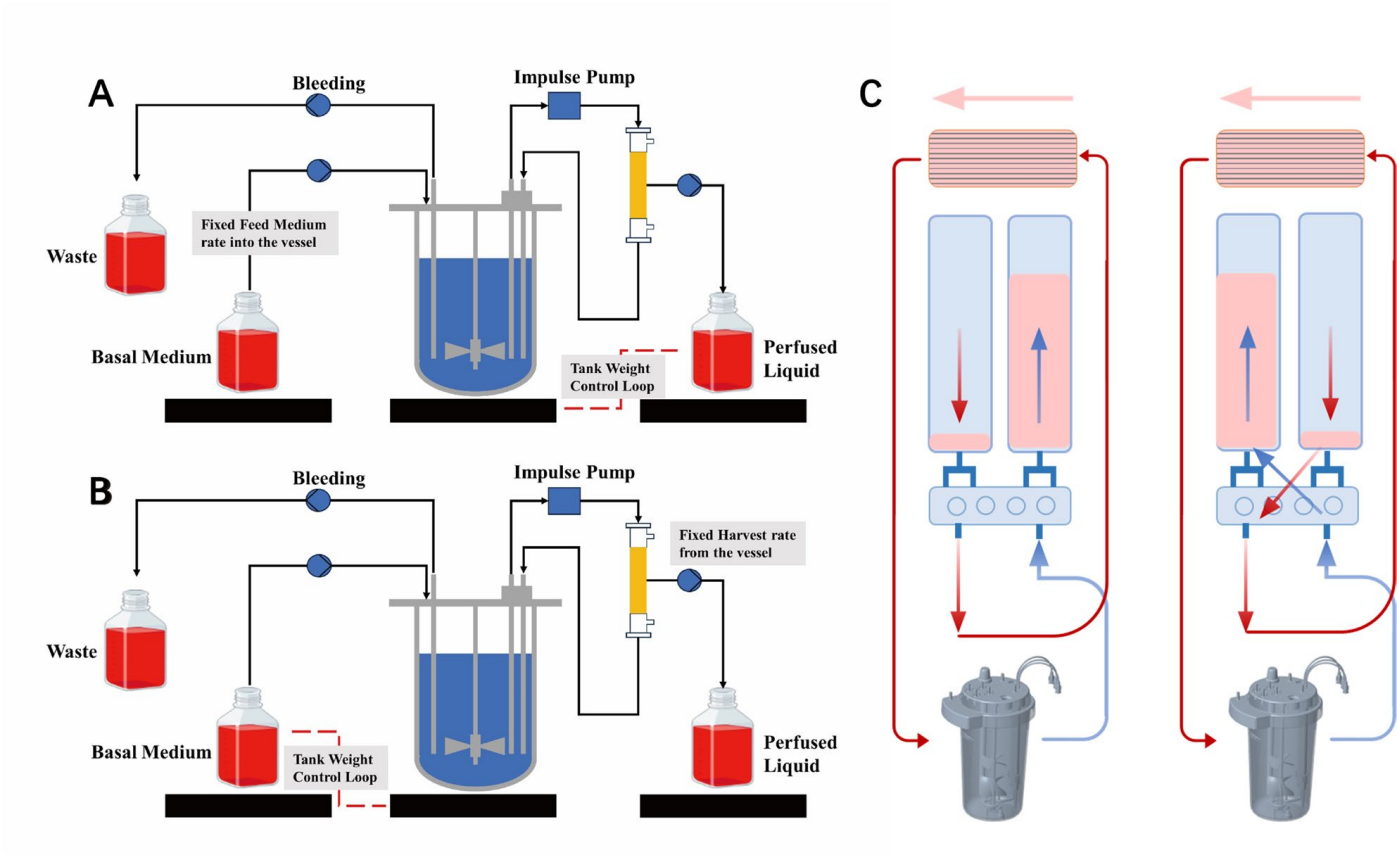
Perfusion culture mode (**A** is tank weight control mode with constant harvest rate; **B** is tank weight control mode with constant feed rate; **C** is a schematic diagram of the working principle of the pulse pump)

This study develops an Impulse Tangential-flow Filtration (ITF) system based on TFF, as depicted in Fig. 2 The ITF system incorporates a pulsating low-shear recirculation pump, which is synergistically coupled with an external vacuum pump to facilitate the injection of sterile air. This configuration enables the system to achieve alternating positive and negative pressure cycling for efficient power transfer. The operational integrity is further enhanced by a valve group control mechanism, ensuring both low-shear and low-power conditions are maintained throughout the process (Fig. 2C). During the positive pressure phase, sterile air is injected to push the culture medium through the hollow fiber membrane, creating the tangential flow necessary for cell retention. The subsequent negative pressure phase generates a transient suction that reverses the flow direction, creating a “pulse” effect. This pulsating mechanism ensures that both low-shear and low-power conditions are maintained throughout the intensification process, preventing the high-velocity hotspots typically associated with continuous peristaltic pumping. It also features the ability to perform air backflushing, which prevents filter cake formation, extends the membrane’s lifespan, and ensures system stability. In the perfusion mode, fresh culture medium is continuously pumped from the Basal Medium bottle into the tank. ITF can pump the spent culture medium in the tank into Perfused Liquid while trapping the cells in the tank, and at the same time discharge the cells that have broken and settled at the bottom through the pump into the waste bottle. The system monitors the weight of Basal Medium, tank and Perfused Liquid in real time through a control system developed on the basis of a Siemens S7-1200 PLC, which allows tank weight control mode based on a constant replenishment rate and tank weight control mode based on a constant harvest rate.

### Oxygen mass transfer measurement of SUB

To comprehensively investigate the oxygen mass transfer capability of the bioreactor, the volumetric oxygen transfer coefficient ${k}_{L}a$ of the SUB equipped with two layers of elephant ear impellers under different operating conditions was determined using the dynamic method (Kazemzadeh et al. 2020). The specific experimental conditions are detailed in Table 1.

**Table 1:** Experimental condition for ${k}_{L}a$ measurement

Experimental condition	Unit	Value
Aeration rate	mL/min	30–150
Stirring speed	rpm	60–180
Volume	mL	200, 400

Throughout the experiment, the temperature of the bioreactor was maintained at 37 °C, and the pressure was kept at 0.05 MPa. Initially, the bioreactor was deoxygenated by introducing nitrogen into the bioreactor. When the dissolved oxygen level dropped to a lower level, air was then introduced into the bioreactor, and the change in dissolved oxygen (DO) over time was recorded. The ${k}_{L}a$ was calculated by Eq. (1).

1 $$\begin{array}{c}{k}_{L}a\left(t-{t}_{0}\right)=\mathrm{ln}\left(\frac{{DO}^{\mathrm{*}}-{DO}_{{t}_{0}}}{{DO}^{\mathrm{*}}-{DO}_{t}}\right)\end{array}$$

Where ${DO}^{*}$ is the saturated dissolved oxygen in this state, ${DO}_{t}$ is the dissolved oxygen value at moment t, ${t}_{0}$ is the initial time, and ${DO}_{{t}_{0}}$ is the dissolved oxygen value at the initial moment.

### CFD modeling of SUB

#### Two-phase flow models

Given the extremely low aeration rates required for the cell culture, the influence of sparged gas on the liquid-phase momentum is minimal. As the global hydrodynamics are predominantly dictated by mechanical agitation (Li et al. 2020), the effect of micro-bubbles on the macroscopic flow field can be reasonably neglected. Therefore, this study employed the Volume of Fluid (VOF) model to simulate the gas-liquid hydrodynamics and track the macroscopic free surface. In this model, the interface is tracked by solving the transport equation for the volume fraction, which is expressed as (Liu et al. 2016; Zhang et al. 2022):

2 $$\begin{array}{c}\frac{\partial\gamma}{\partial t}+({u}\cdot \nabla )\gamma =0\end{array}$$

Where $$ \gamma $$ denotes the partition function, and $$ \gamma =0$$ denotes the absence of gas phase, and $$ \gamma =1$$ denotes the absence of liquid phase, and $$ 0<\gamma <1$$ denotes the presence of gas-liquid phase; $$ {u}$$ is the velocity vector; $$ t$$ is the time.

The gas and liquid phases in the VOF model share a common velocity field and are solved by the momentum equation, the mass and momentum equations are as follows (Liu et al. 2016; Zhang et al. 2022):

3 $$ \begin{array}{c}\frac{\partial \rho }{\partial t}+\nabla \cdot (\rho {u})=0\end{array}$$

4 $$ \begin{aligned}\frac{\partial }{\partial t}\left(\rho {u}\right)+\nabla \cdot \left(\rho {u}{u}\right)& =-\nabla p+\nabla \cdot \left[\mu \left(\nabla {u}+\nabla {{u}}^{{T}}\right)\right]\\&\quad +\rho g+F\end{aligned}$$

Where $$ p$$ is the pressure (p.a.); $$ {g}$$ is the gravitational acceleration (m/s^2^); $$ {F}$$ is the gas-liquid surface tension, which is described by the Brackbill model; and $$ \rho $$and $$ \mu $$ are the weighted average values of density and viscosity in the gas-liquid phases, respectively, which are expressed by Eqs. (5) and (6). Additionally, T denotes the transpose matrix of the velocity gradient tensor.

5 $$ \begin{array}{c}\rho =\gamma {\rho }_{g}+\left(1-\gamma \right){\rho }_{l}\end{array}$$

6 $$ \begin{array}{c}\mu =\gamma {\mu }_{g}+\left(1-\gamma \right){\mu }_{l}\end{array}$$

Where ${\rho }_{g}$ and ${\rho }_{l}$ are the density of the gas and liquid phases; ${\mu }_{g}$and ${\mu }_{l}$ are the viscosity of the gas and liquid phases.

#### Turbulence models

The turbulence model was chosen as the Renormalization Group (RNG) $k-\epsilon$ model. Derived from rigorous statistical techniques of the renormalization group theory, the RNG model refines the standard $k-\epsilon$ model by explicitly accounting for low-Reynolds-number effects. This makes it significantly more robust and accurate for characterizing the transitional flows and strong swirling flows encountered in the stirred tank (Aubin et al. 2004). The transport equations for the turbulence generation term and the turbulence dissipation rate are expressed as follows (Yakhot and Orszag 1986):

7 $$ \begin{array}{c}\frac{\partial \left(\rho k\right)}{\partial t}+\nabla \cdot \left(\rho {u}k\right)=\nabla \cdot \left[\left(\mu +\frac{{\mu }_{t}}{{\sigma }_{k}}\right)\nabla k\right]+{P}_{k}+{P}_{kb}-\rho \epsilon\end{array}$$

8 $$\begin{aligned} \frac{\partial \left(\rho \epsilon \right)}{\partial t}+\nabla \cdot \left(\rho {u}\epsilon \right)&=\nabla \cdot \left[\left(\mu +\frac{{\mu }_{t}}{{\sigma }_{\epsilon RNG}}\right)\nabla \epsilon \right]\\& \quad +\frac{\epsilon }{k}[{C}_{\epsilon 1RNG}({P}_{k}+{P}_{\epsilon b})]-{C}_{\epsilon 2RNG}\end{aligned}$$

Where $$ k$$ is the turbulent kinetic energy; $$ \epsilon $$ is the turbulent dissipation rate, which can be calculated by Eq. (9); $$ {\sigma }_{k}$$, $$ {\sigma }_{\epsilon RNG}$$, $$ {C}_{\epsilon 1RNG}$$ and $$ {C}_{\epsilon 2RNG}$$ are constants; $$ {P}_{kb}$$ and$$ {P}_{\epsilon b}$$ are the effects of buoyancy; $$ {\mu }_{t}$$ is the turbulent viscosity; and $$ {P}_{k}$$ is the turbulence generation term due to viscous forces.

9 $$ \begin{array}{c}\epsilon =\mu \cdot \left[\nabla {u}+{\left(\nabla {u}\right)}^{T}\right]:\nabla u\end{array}$$

In Eq. 9, the symbol “:” denotes the double-dot product (double contraction) of the velocity gradient tensors.

The shear in the flow field is a vector quantity; however, it is not ideal to use vectors to represent the magnitude of shear. The Shear Strain Rate (SSR, s^−1^) is a scalar quantity that captures the change in velocity gradients within the flow field, and it is expressed as (Li et al. 2020):

10 $$ \begin{aligned}&SSR=\\&\quad \sqrt{2\left[{\left(\frac{\partial {u}_{x}}{\partial x}\right)}^{2}+{\left(\frac{\partial {u}_{y}}{\partial y}\right)}^{2}+{\left(\frac{\partial {u}_{z}}{\partial z}\right)}^{2}\right]+{\left(\frac{\partial {u}_{x}}{\partial x}+\frac{\partial {u}_{y}}{\partial y}\right)}^{2}+{\left(\frac{\partial {u}_{x}}{\partial x}+\frac{\partial {u}_{z}}{\partial z}\right)}^{2}+{\left(\frac{\partial {u}_{y}}{\partial y}+\frac{\partial {u}_{z}}{\partial z}\right)}^{2}}\end{aligned}$$

#### Simulation details

This study conducted a comprehensive investigation into the flow field characteristics of the SUB under different operating conditions using CFD technology, as detailed in Table 2. The 3D model of the SUB was constructed using Creo 7.0 software (PTC, Boston, MA, USA), and tetrahedral unstructured meshing was performed with ANSYS ICEM software (ANSYS Inc., Canonsburg, PA, USA). Mesh refinement was applied to the wall and rotating domains, with a minimum mesh size of 0.5 mm and a maximum of 10 mm.

**Table 2:** CFD simulation condition for SUB

CFD Simulation condition	Unit	Value
Impeller Type	–	EE-EE, EE-RB
Stirring speed	rpm	60–240
Volume	mL	90, 200, 480

EE-EE refers to double Elephant Ear impellers; EE-RB refers to the combination of Elephant Ear and Ribbon impellers

To ensure the accuracy and reliability of the numerical results, a grid independence study was performed using four different mesh resolutions: 1.05, 1.60, 2.30, and 3.10 million cells (Supplementary Figure S1). Key hydrodynamic parameters, including impeller torque, average velocity, average SSR, and average TED, were monitored across these scales. As illustrated in the mesh convergence plots, the variation in these parameters became negligible once the mesh count reached 2.30 million. Specifically, the deviation in torque and average SSR between the 2.30 million and 3.10 million grids was less than 1.5%, indicating that the simulation results were independent of the grid resolution. Consequently, a mesh count of approximately 2.30 million was selected for all subsequent computations. The steady-state solution of the SUB model was carried out on a Sugon server equipped with 96 processors, utilizing the ANSYS CFX 15.0 solver (ANSYS Inc., Canonsburg, PA, USA). The liquid phase was set to water and the gas phase to air, with a free surface at the interface between the two phases. These specific working volumes (90 mL, 200 mL, and 480 mL) were selected to evaluate the impellers’ impact on the flow field under distinct operational conditions: 90 mL represents a state where the bottom impeller is partially submerged, 200 mL corresponds to the maximum volume for a single-impeller configuration, and 480 mL reflects the maximum working capacity. The convergence residual was set to 10^−4^, and the solver process monitored the torque of the impellers, with convergence considered achieved upon stabilization.

### Cell perfusion culture

#### CHO-K1 cell line

Recombinant Chinese hamster ovary CHO-K1 cell line (supplied by Alit Biotech (Shanghai) Co., Ltd) was used to produce antibody protein drugs. The initial cell volume of the reactor after inoculation was 5 × 10^5^ cells/mL, and the working volume was 300 mL. The bioreactor featured an EE-EE impeller configuration operating at an initial stirring speed of 150 rpm, with the temperature maintained at 36.5 °C. The DO was regulated at 40% saturation through a combination of aeration (air and pure oxygen) and agitation. Specifically, oxygen was introduced once the air flow rate reached its threshold of 3 mL/min(14.4 vvd), after which the air flow remained constant. In addition, constant headspace overlay aeration was maintained at 100 mL/min (480 vvd) using air to facilitate CO_2_ stripping. The pH was maintained at 7.1 with a deadband of 0.1, automatically controlled via the addition of CO_2_. The cultivation period lasted for 35 days. Perfusion culture was initiated on the third day of cultivation to timely remove the old medium and product from SUB, aiming to increase product yield and reduce the risk of product degradation. The perfusion rate was increased with the rise in cell density, reaching a maximum of 2 vvd. Low-temperature culture is a commonly employed method in CHO cell culture, which can prevent premature apoptosis to maintain cell viability and promote the expression of antibody protein analogues (Chen et al. 2011; Coronel et al. 2020). Perfusion culture was initiated on the third day of cultivation (when the expected cell density reached approximately 5 × 10^6^ cells/mL) to timely remove the old medium. When the cell density reached 8.00 × 10^7^ cells/mL, the culture temperature was lowered to 32 °C. Cell density and cell viability were determined using a cell counter and trypan blue assay. In this study, the perfusion system was operated using the tank weight control mode based on a constant harvest rate. To maintain the target viable cell density and remove non-viable cells, cell bleeding was performed manually on a daily basis starting from day 10 by withdrawing a calculated volume of cell suspension directly from the reactor.

#### Expi293 cell line

The Expi293 cell line (supplied by Alit Biotech (Shanghai) Co., Ltd), which carries adeno-associated virus and virus-related genes, is commonly used in the production of viral vaccines. The initial cell inoculation density was 8.00 × 10^5^ cells/mL with a working volume of 330 ml. Stirring was conducted using an EE-EE impeller configuration at an initial stirring speed of 200 rpm, with the culture temperature maintained at 37℃. The DO was controlled at 30%, dependent on aeration and stirring speed, with a maximum stirring speed of 350 rpm. The cultivation period is 14 days, and when the cell density reaches 4 × 10^6^ cells/mL, the perfusion rate starts at 0.5 vvd and increases with the cell density, with a maximum perfusion rate of up to 4 vvd.

## Results and discussion

### Oxygen mass transfer

**Fig. 3:**
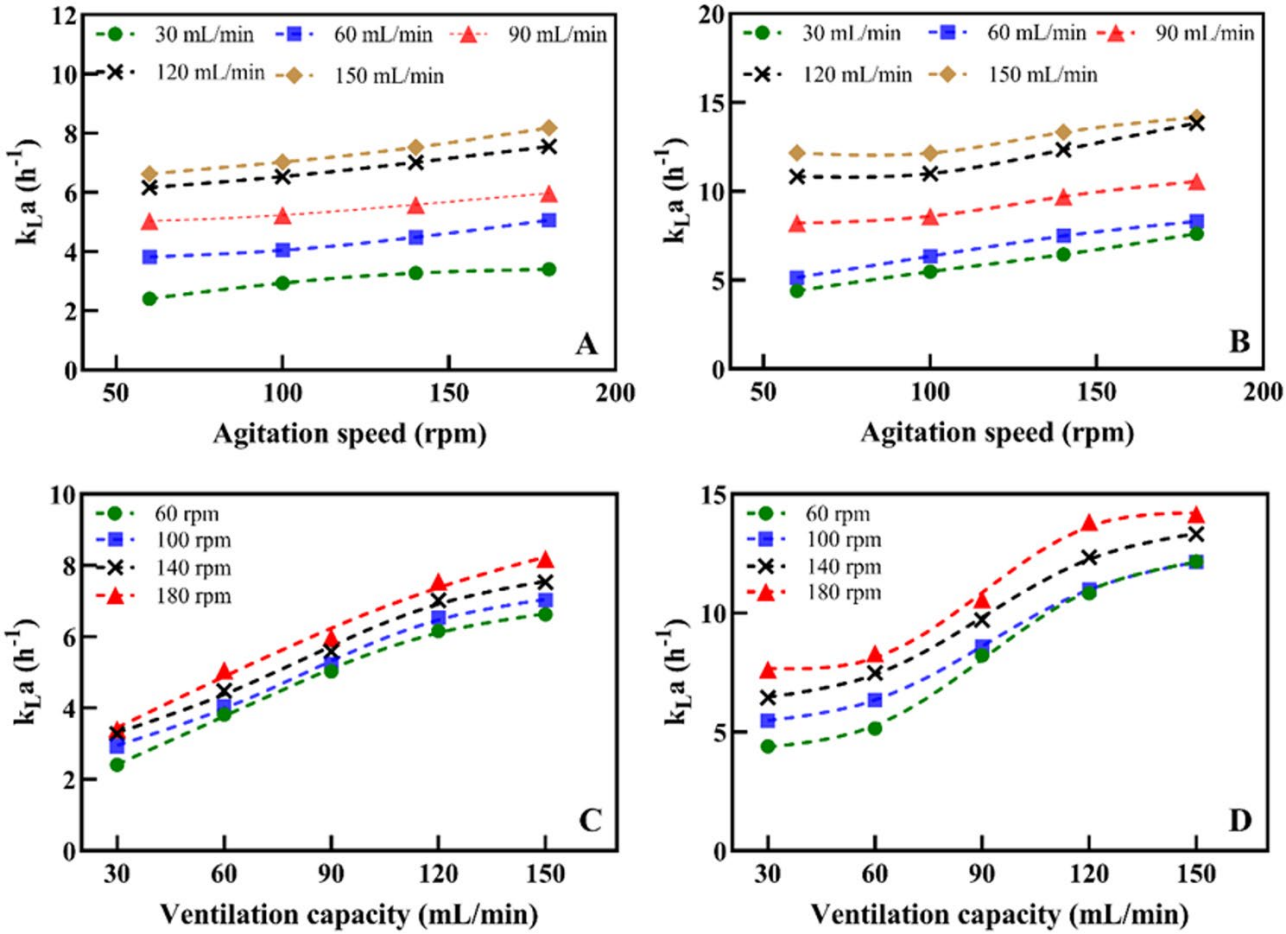
The measurements result of $$ {k}_{L}a$$ in SUB (**A** and **B** are the change of $$ {k}_{L}a$$ with stirring speed at different aeration rates for working volumes of 400 mL and 200 mL, respectively; **C** and **D** are the variation of $$ {k}_{L}a$$ with aeration rate at different stirring speeds for working volumes of 400 mL and 200 mL, respectively)

The dynamic method was employed to determine the $$ {k}_{L}a$$ inside the SUB (Fig. 3). Overall, $$ {k}_{L}a$$ increased significantly in response to elevated stirring speeds and aeration rates. Mechanistically, increasing the aeration rate directly expands the gas-liquid interfacial area by introducing a higher volume of dispersed gas. Under low-aeration conditions, the system is fundamentally gas-limited; therefore, the increase in stirring speed exerts only a marginal effect on mass transfer. Conversely, under high-aeration conditions (> 90 mL/min, 324vvd), high-speed agitation (exceeding 100 rpm) becomes essential. At this threshold, the kinetic energy from the impellers is sufficient to overcome the surface tension of the gas bubbles, shearing them into smaller micro-bubbles. This secondary dispersion radically increases the interfacial area and extends gas residence time, preventing bubble coalescence and channelization. The specific oxygen uptake rate of mammalian cells ranges from 0.5 × 10^−10^ to 8.0 × 10^−10^ mmol/cell/h. For a reactor system containing 10^7^ cells/mL, the required $$ {k}_{L}a$$ ranges from 3 to 50 h^−1^ under sterile air conditions, and from 0.5 to 8 h^−1^ under pure oxygen conditions. Biogenstar has consistent mass transfer performance with the mainstream AMBR bioreactors on the market (Xu et al. 2017a; Li et al. 2018; Keller et al. 2022; Anand et al. 2024). Therefore, to avoid excessive energy input, the $$ {k}_{L}a$$ of animal cell culture reactors should be maintained within the range of 1–15 h^−1^ to ensure balanced oxygen input and carbon dioxide removal (Nienow 2006). In this study, the overall $$ {k}_{L}a$$ of the SUB ranges from 2 to approximately 15 h^−1^ (Xing et al. 2009), which is capable of meeting the highest oxygen consumption requirements for mammalian cell cultures such as CHO cells (Pérez-Rodriguez et al. 2022).

### Flow pattern and velocity distribution

**Fig. 4:**
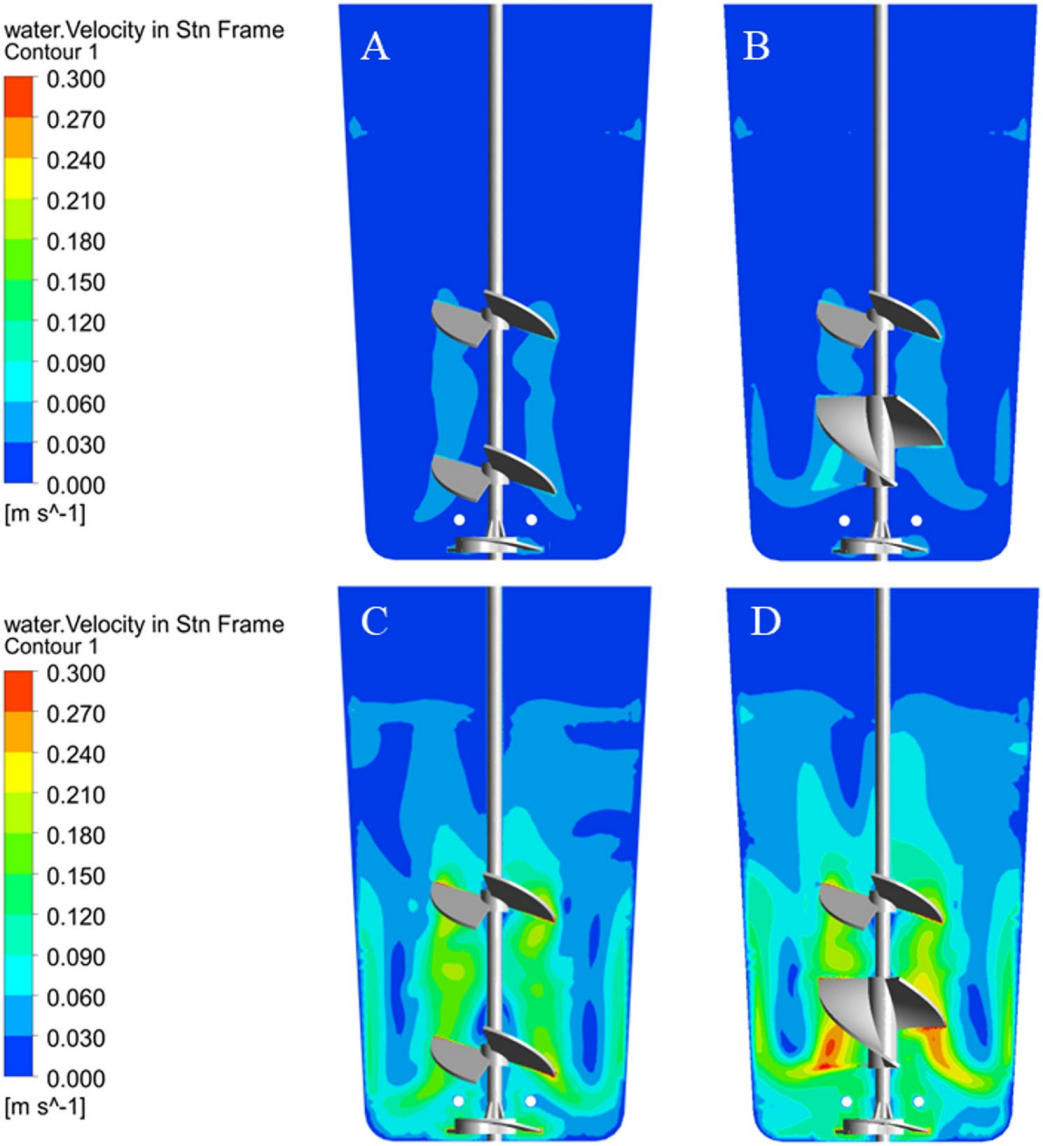
The velocity contour plot of SUB (**A** and **B** are the velocity distributions of the EE-EE impeller and the EE-RB impeller at 60 rpm; **C** and **D** are the velocity distributions of the EE-EE impeller and the EE-RB impeller at 240 rpm)

**Fig. 5:**
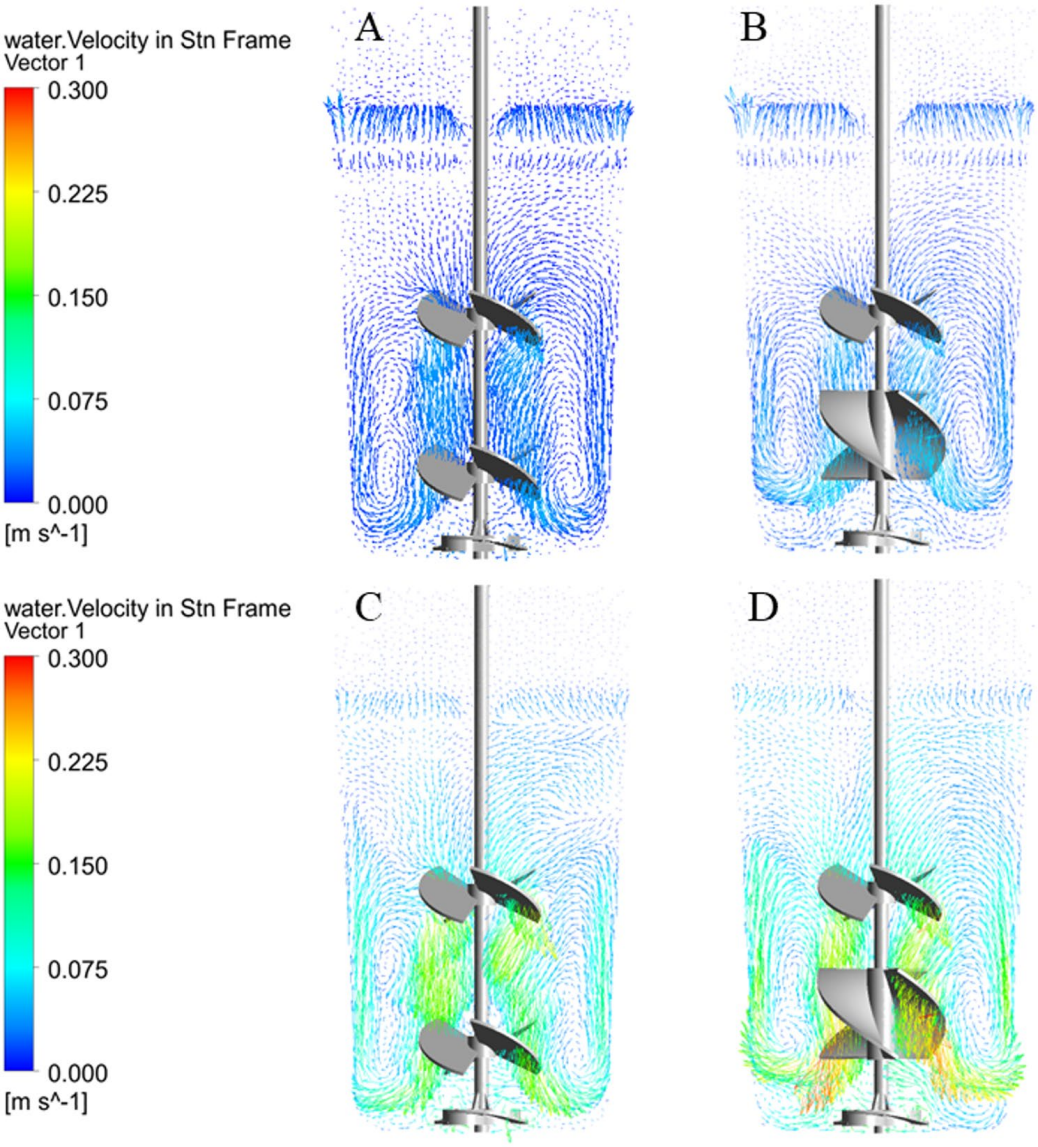
The velocity vector plot of SUB (**A** and** B** are the velocity vector of the EE-EE impeller and the EE-RB impeller at 60 rpm;** C** and** D** are the velocity vector of the EE-EE impeller and the EE-RB impeller at 240 rpm)

The velocity contour (Fig. 4) and vector plots (Fig. 5) reveal the distinct hydrodynamic mechanisms driving fluid homogenization under the Elephant Ear-Elephant Ear (EE-EE) and Elephant Ear-Ribbon (EE-RB) configurations. Both EE and RB are axial-flow impellers; however, their geometric differences dictate specific kinetic energy dissipation patterns. The EE-RB combination established a dominant macro-scale axial bulk flow. Because the continuous helical structure of the RB impeller provides a larger sweeping surface area, it yields a higher volumetric discharge capacity than the pitched blades of the EE impeller. While the EE-RB combination offers superior global axial mixing at equivalent speeds, its dispersed energy transfer reduces the localized, high-velocity jetting needed to generate strong secondary circulation loops at the absolute bottom of the vessel. Consequently, the EE-EE configuration, despite a slightly lower global discharge rate, provides more focused kinetic energy to the vessel floor, mitigating the risk of cell sedimentation during prolonged cultures. Specifically, the EE impeller predominantly generates a down-flow axial pattern.

### Shear strain rate

**Fig. 6:**
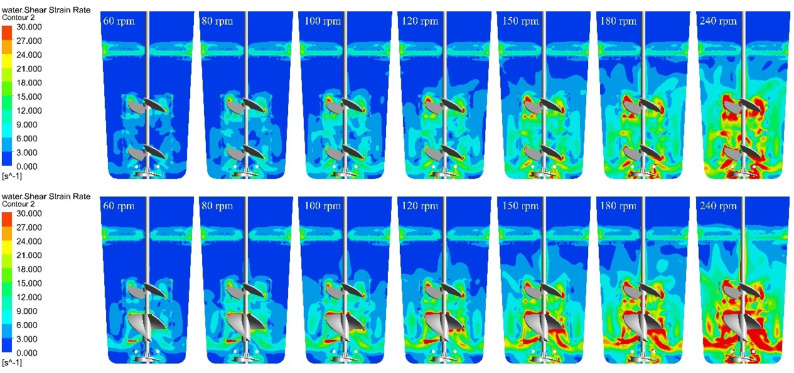
The SSR distribution of SUB (Top is shear rate distribution of 480 mL under the EE-EE impeller combination with different agitation speeds; Bottom is shear rate distribution of 480 mL under the EE-RB impeller combination with different agitation speeds)

The sensitivity of animal cell culture to shear stress necessitates a detailed evaluation of the shear environment within the reactor. As illustrated in Fig. 6, localized zones of elevated SSR are predictably confined to the immediate vicinity of the impellers, sparger, and vessel walls, scaling linearly with agitation speed. While the average SSR inside the bioreactor decreases slightly with increasing loading volume, it remains fundamentally driven by impeller kinematics, with the bottom rotor generating the highest local SSR, followed by the RB and elephant ear EE impellers.

**Fig. 7:**
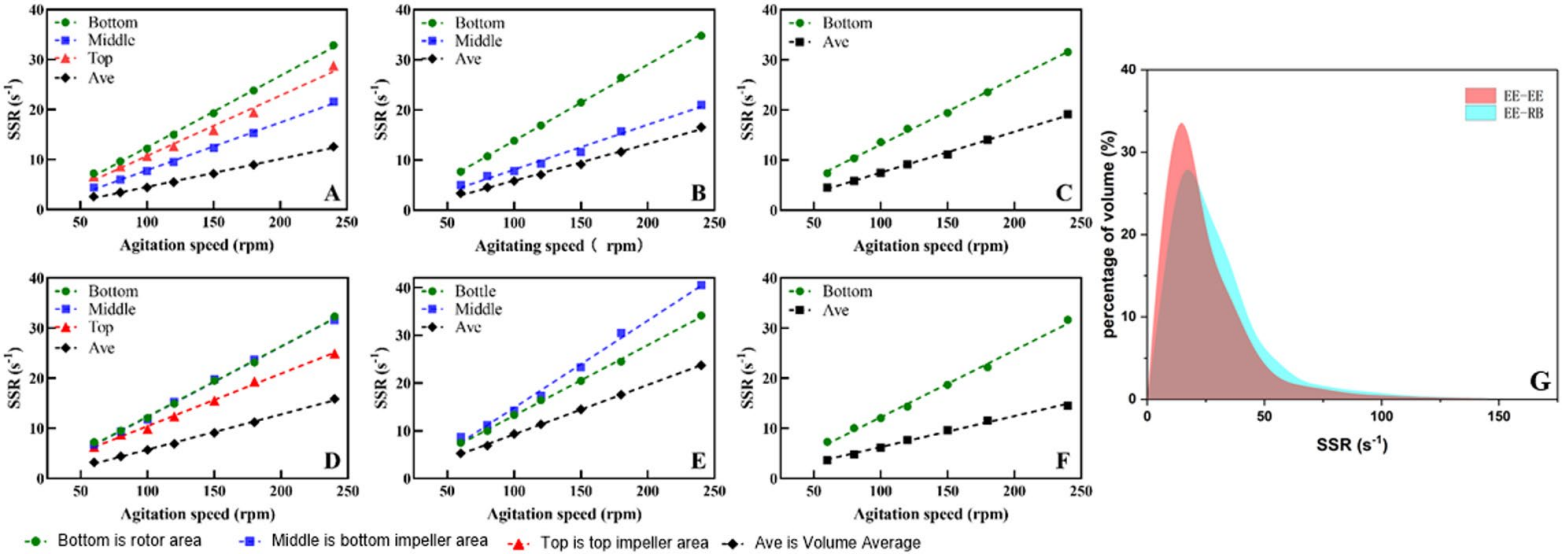
The quantitative plot of SSR (**A**–**C** are the shear rate at different agitation speeds under the volume of the EE-EE impeller combination of 480, 200 and 90 mL respectively; **D**–**F** are the shear rate at different agitation speeds under the volume of the EE-RB impeller combination of 480, 200 and 90 mL respectively; **G** is the fluid volume percentage of two types of impellers combined at 240 rpm under different SSR levels. Bottom is rotor area; Middle is bottom impeller area; Top is top impeller area; Ave is the average shear rate of SUB; Note: For the 90 mL and 200 mL working volumes in panels **B**, **C**, **E**, and **F**, Top and/or Middle data points are excluded because the corresponding impellers were not fully submerged and were located in the gas phase)

To quantitatively validate the hydrodynamic safety of the entire vessel, the volumetric SSR distribution was analyzed (Fig. 7G). The probability density profiles confirm that both impeller configurations maintain an overwhelmingly gentle shear environment. The EE-EE configuration exhibits a highly concentrated distribution, peaking sharply at approximately 15 s^−1^ (representing ~ 33% of the total fluid volume). In contrast, the EE-RB configuration displays a slightly broader distribution, peaking near 20 s^−1^ (~ 27% volume fraction) with a marginally extended tail. This broader dissipation profile mechanistically aligns with the ribbon impeller’s larger continuous surface area, which distributes kinetic energy across a wider sweeping volume rather than concentrating it at the blade tips.

Crucially, despite these geometrical differences, the volumetric analysis demonstrates that > 95% of the total bioreactor volume in both configurations operates at an SSR well below 50 s^−1^. This volumetric safety margin is critical for bioprocess intensification. Empirical literature establishes that ultimate localized SSR damage thresholds are approximately 10^5^ s^−1^ for CHO cells (Strobl et al. 2021) and 400 s^−1^ for stem cells (Arora et al. 2019). Even highly sensitive cell lines like BHK-21 require the average SSR strictly below 92 s^−1^ to avert structural and metabolic disruption; at 50 s^−1^, they can metabolize normally, though energy allocation for membrane repair begins to shift (Goodwin et al. 1993).

Because the global average SSR remains strictly below 25 s^−1^ and the volumetric distribution lacks any significant high-shear fractions, the SUB establishes a fundamentally “hydrodynamically safe” regime. By avoiding localized hydrodynamic hotspots, this profile prevents viscous drag forces from deforming cellular lipid bilayers beyond their elastic limits. Consequently, the bioreactor accommodates the vigorous agitation required for high-density perfusion without inducing mechanical cell death. As summarized in Table 3, the SUB achieved superior $$ {k}_{L}a$$ at lower aeration rates while maintaining significantly lower SSR and P/V compared to benchmark reports. In conventional microbial fermentation, scale-up criteria are typically based on maintaining a constant $$ {k}_{L}a$$ impeller tip speed, or *P/V*. However, because mammalian cells lack a protective cell wall, they exhibit heightened mechanosensitivity, rendering these traditional scalar approaches inadequate. Consequently, the precise, quantitative characterization of the shear environment—such as our volumetric distribution analysis and previously proposed three-dimensional shear space models (Li et al. 2020; Teng et al. 2021) emerges as a critical, non-negotiable requirement for scalable mammalian cell cultivation.

**Table 3:** SUB characterized by CFD method

Single-use bioreactor	Volume (L)	Stirring speed (rpm)	Aeration (vvm)	$$ {{k}}_{{L}}{a}$$ (h^−1^)	TED (10^−3^ m^2^/s^3^)	SSR (s^−1^)	$$ {P}/{V}$$ (W/m^3^)	$$ {{t}}_{95}$$ (s)	References
This study	0.2–0.48	60–240	0.0625–0.75	2–15	0.2–5.3	< 25	0.1–10	1–10	
Ambr250 (RT)		200–800	0.001–0.08	0.18–7.9	1–90		1.3–86.7		Li et al. (2018)
Ambr250 (EE)		200–600		0.8–3.7			8-36.9	3–20	Anand et al. (2024)
Flexible bag bioreactor	10	50–200	0.1–0.2	0.3–6	6.5–1352	70–1550	38–8220	2–13.8	Mishra et al. (2021)
CellReady 3 L	1.5–2.5	80–120		5.3–9.8			2.4–37	3.9–33.4	Kaiser et al. (2011)
Biostat 5 L	4.5	600–700	1.5-2			600–3000	100–5000		Vlaev et al. (2018)

### Turbulence eddy dissipation

**Fig. 8:**
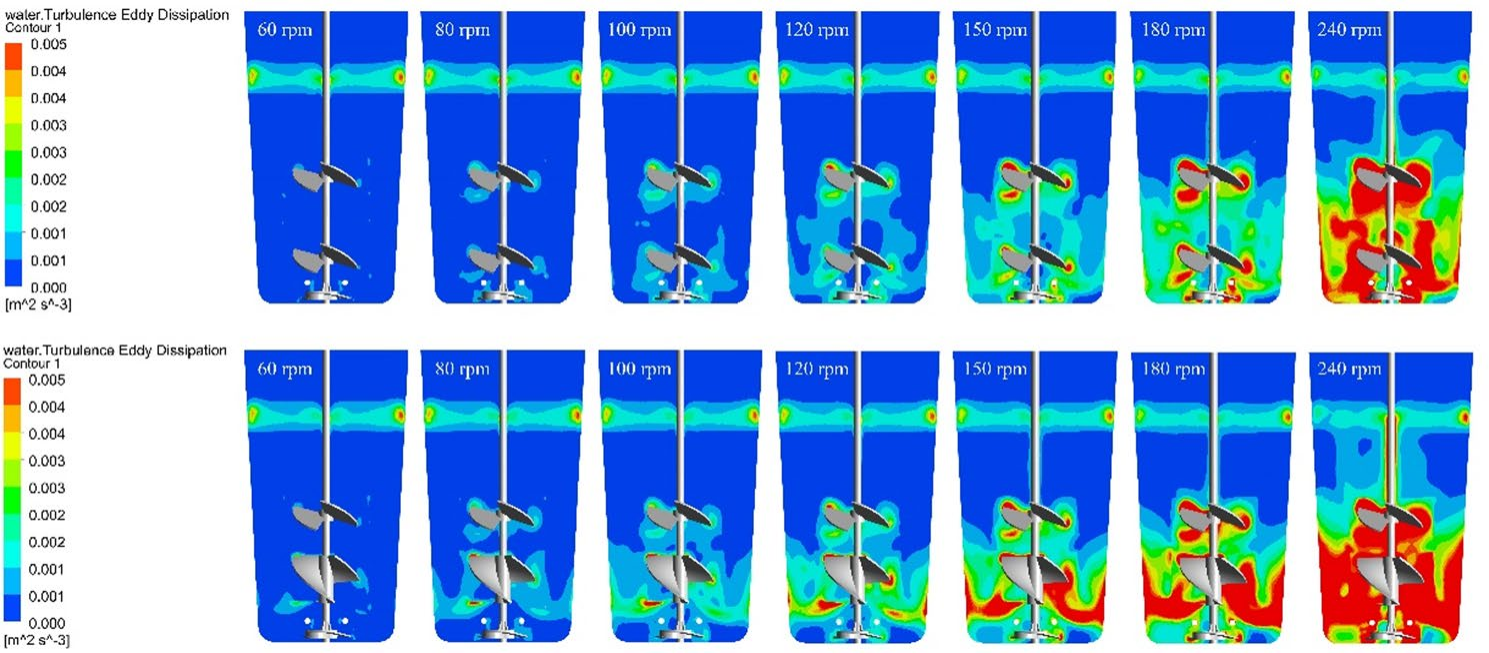
The Turbulence Eddy Dissipation of SUB (Top is TED distribution of 480 mL under the EE-EE impeller combination with different agitation speeds; Bottom is TED distribution of 480 mL under the EE-RB impeller combination with different agitation speeds)

Figure 8 illustrates the distribution of TED at various stirring speeds for the two impeller combinations at a working volume of 480 mL. Elevated TED zones are primarily concentrated near the impellers, air sparger, and vessel walls, intensifying with increased agitation. Because the formulas for both SSR and TED are derived from velocity gradients, their spatial distributions exhibit distinct similarities. For the EE-EE impeller configuration, the maximum localized TED ranges from 1.25 × 10^−4^ to 3.13 × 10⁻^3^ m^2^/s^3^ across the tested stirring speeds. Due to its larger continuous blade area and higher volumetric discharge, the RB impeller generates higher local TED than the elephant EE impeller. Consequently, the maximum localized TED for the EE-RB configuration is slightly higher, ranging from 1.96 × 10^−4^ to 5.34 × 10^−3^ m^2^/s^3^.

Fundamentally, TED is a microenvironmental variable driven by the velocity field and kinematic viscosity, making it an excellent metric for quantifying localized hydrodynamic forces within a non-homogeneous flow field. Similar to shear stress, mammalian cells exhibit specific physiological tolerance limits for TED. It is crucial, however, to differentiate between the global average energy input and these localized extremes. For CHO cells, literature indicates that when cells are exposed to localized TED values reaching or exceeding 100 m^2^/s^3^ (equivalent to an extreme localized power dissipation of 10^5^ W/m^3^), they undergo growth arrest or catastrophic lysis (Godoy-Silva et al. 2009; Mishra et al. 2021). To provide a robust safety margin against frequent exposure to high-energy micro-zones, the average TED environment in a mammalian cell bioreactor should be strictly maintained below 10 m^2^/s^3^ (equivalent to 10^4^ W/m^3^) (Eibl et al. 2009). Mechanistically, this critical safety threshold of TED can be fundamentally elucidated through its relationship with the Kolmogorov length scale (λ). In isotropic turbulence theory, λ represents the size of the smallest turbulent eddies where kinetic energy is ultimately dissipated into heat, calculated as $$ \lambda ={({v}^{3}/\epsilon )}^{1/4}$$ (where $$ v$$ is the kinematic viscosity and $$ \epsilon $$ is the local TED). For mammalian cells like CHO and HEK293, which typically possess a diameter of 15–20 μm, fatal hydrodynamic damage is predominantly induced when the size of these energy-dissipating eddies becomes smaller than the cells themselves. Under such conditions, cells are subjected to severe, localized pressure fluctuations and mechanical stretching across their surfaces. By strictly maintaining the average TED below 10 m^2^/s^3^, the corresponding Kolmogorov eddy size remains approximately 18 μm or larger. This physical condition ensures that the micro-eddies are generally larger than the individual cells, allowing the cells to predominantly translate and rotate along with the fluid streamlines rather than being torn apart by microscopic spatial velocity gradients. This intrinsic hydrodynamic mechanism explains the preservation of cellular lipid bilayer integrity and the consequent high viability observed in our high-density perfusion cultures.

### Power consumption

**Fig. 9:**
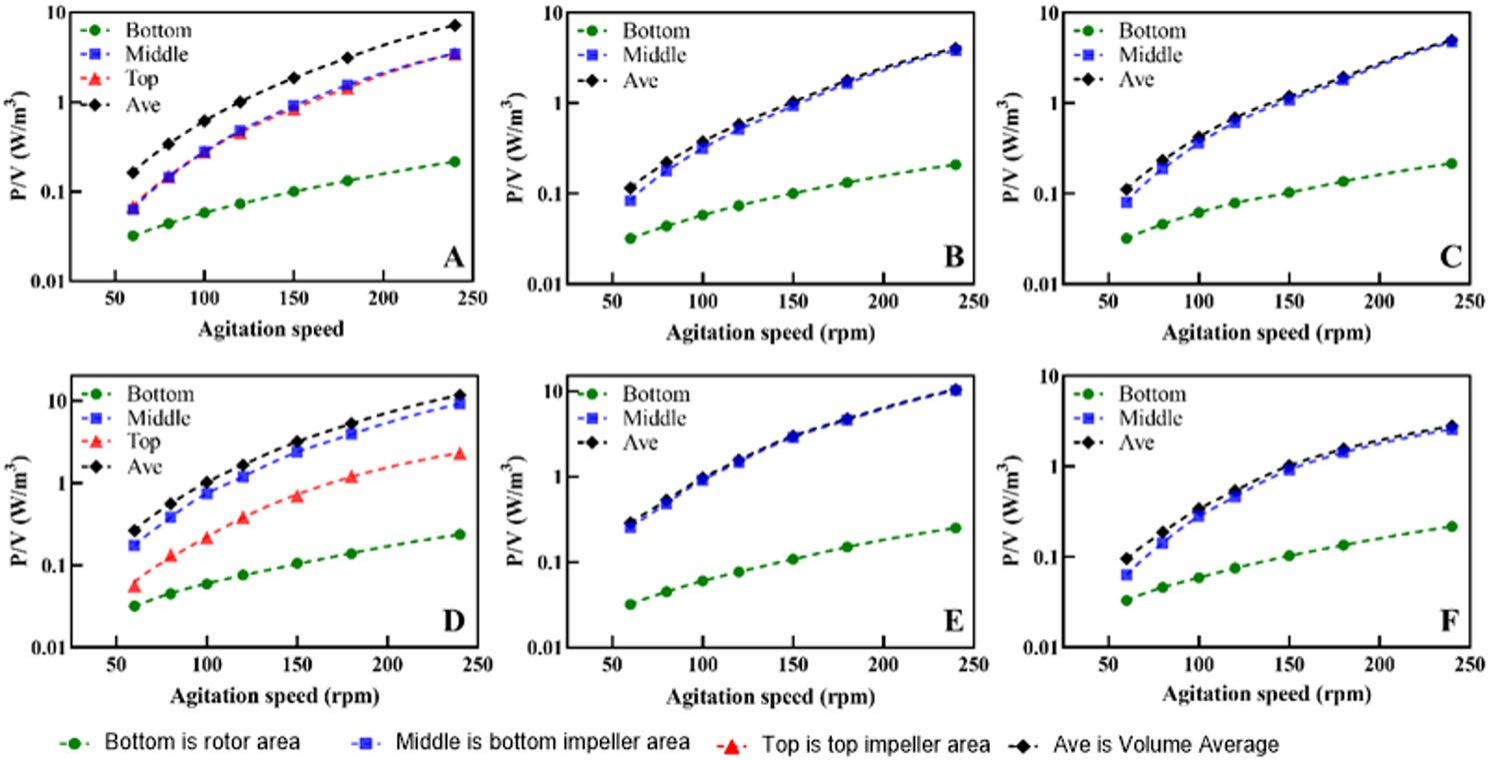
The power per unit volume $$ P/V$$ of SUB (**A**–**C** are the $$ P/V$$ at different agitation speeds under the volume of the EE-EE impeller combination of 480, 200 and 90 mL respectively; **D**–**F** are the $$ P/V$$ at different agitation speeds under the volume of the EE-RB impeller combination of 480, 200 and 90 mL respectively. Bottom is rotor area; Middle is bottom impeller area; Top is top impeller area; Ave is the average $$ P/V$$ of SUB; Note: The Y-axis (P/V) is presented on a Log_10_ scale)

**Fig. 10:**
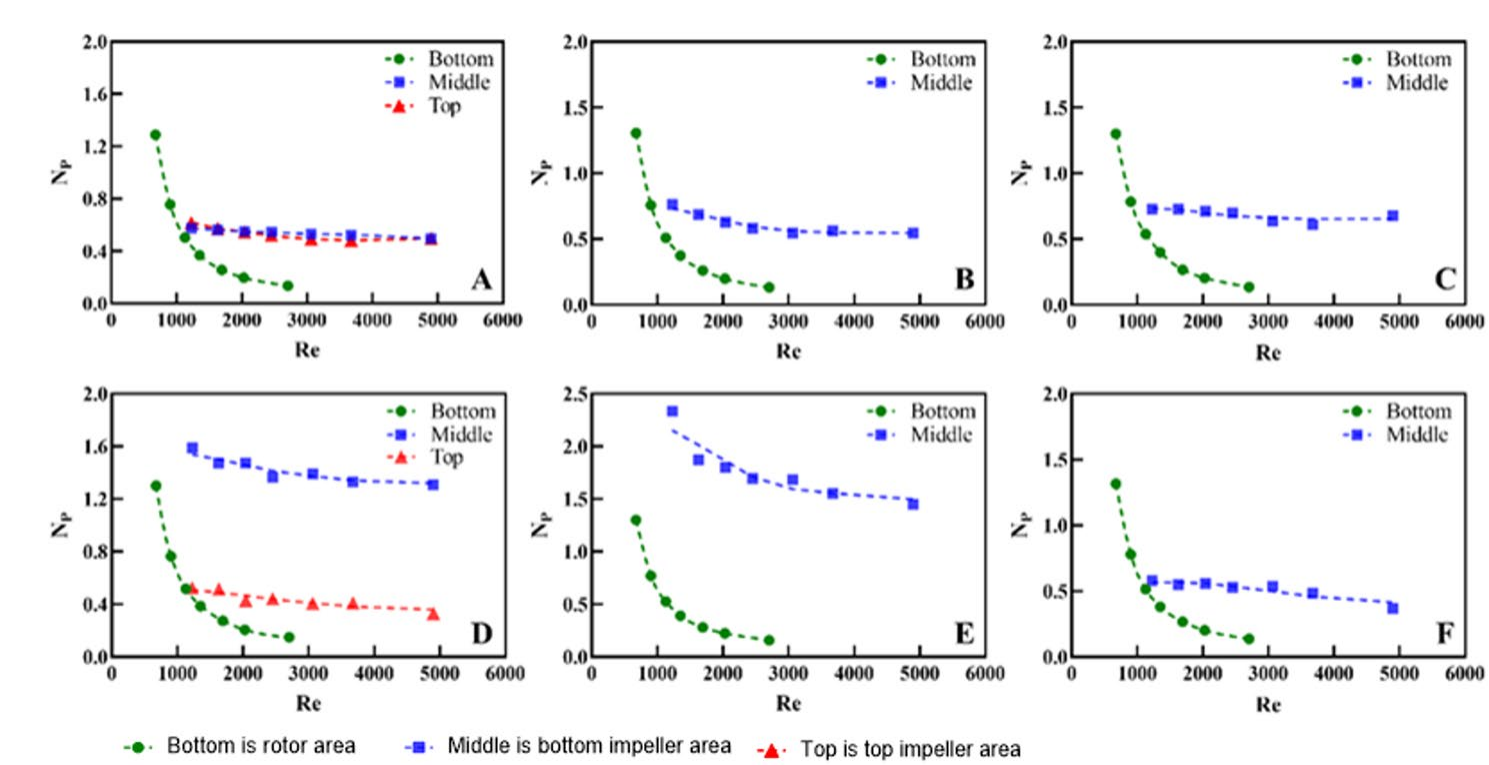
The $$ {N}_{P}$$ under different Re of SUB (**A**–**C** are the $$ {N}_{P}$$ at different Re under the volume of the EE-EE impeller combination of 480, 200 and 90 mL respectively; **D**–**F** are the $$ {N}_{P}$$ at different Re under the volume of the EE-RB impeller combination of 480, 200 and 90 mL respectively. Bottom is rotor area; Middle is bottom impeller area; Top is top impeller area)

The total power input *P* (defined as the sum of the power drawn by all active impellers and the magnetic rotor, W) and the power input per unit volume *P/V* (W/m^3^) are crucial indicators for evaluating the energy consumption and stress environment of stirred bioreactors. We obtained the torques for each impeller through CFD simulations and calculated the $$ P$$ and $$ {N}_{P}$$, with the results shown in Figs. 9 and 10. As can be seen in Fig. 9, the power input for each impeller and the total power input of the bioreactor increase exponentially with the stirring speed. The liquid volume does not significantly affect the $$ P$$ of the stirring impeller, and the $$ P$$ remains stable as long as the impeller is fully submerged (Fig. 9A). In the EE-EE impeller configuration, the stirring power of the two EE impellers is essentially the same, indicating that the flow pattern between the impellers is not significantly affected. The $$ P$$ of the bottom rotor is the lowest, while the $$ {N}_{P}$$ of the RB impeller is significantly higher than that of the EE impeller (Fig. 10). In various operating conditions of this study, the maximum power per unit volume $$ P/V$$ of the SUB system is approximately 10 W/m^3^, with the minimum being about 0.1 W/m^3^. According to Amer et al. (2019), to prevent excessive energy input from causing damage to mammalian cells, the $$ P/V$$ of bioreactors used for mammalian cell culture should be below 50 W/m^3^. In this study, under standard operating conditions (150 rpm and a 300 mL working volume), the specific power input *P/V* was approximately 3 W/m^3^, Therefore, the power input $$ P$$ of this SUB system in this study meets the requirements for mammalian cell culture.

The $$ {N}_{P}$$ of the stirring impeller can be obtained by fitting the data from CFD simulations of different stirring speeds. Figure 10 illustrates the $$ {N}_{P}$$ of the stirring impellers and the bottom rotor under different turbulence intensities (expressed as Reynolds numbers). There is a significant correlation between $$ {N}_{P}$$ and Reynolds numbers (Re), which is consistent in similar geometric configurations (Nienow 2014). The close relationship between $$ {N}_{P}$$ and Re has been validated in similar geometric systems (Delbridge et al. 2023a, b). Under the EE-EE impeller combination condition, the $$ {N}_{P}$$ of the two impellers are close to each other (Fig. 10A), which is about 0.6, which aligns with the findings of Rotondi et al. (Rotondi et al. 2021), indicating the accuracy of the CFD simulation results in this study. The $$ {N}_{P}$$ of the RB impeller is about 1.2 due to the larger blade areas, so the input power of the combined EE-RB impeller is significantly higher than that of the EE-EE impeller combination. When the blades are completely submerged, the $$ {N}_{P}$$ of single-layer and double-layer blades are approximately equal, which indicates that the interference of blade flow pattern is weak under the current configuration, and proves that the selection of blade size and spacing of SUB is reasonable.

### Mixing

**Fig. 11:**
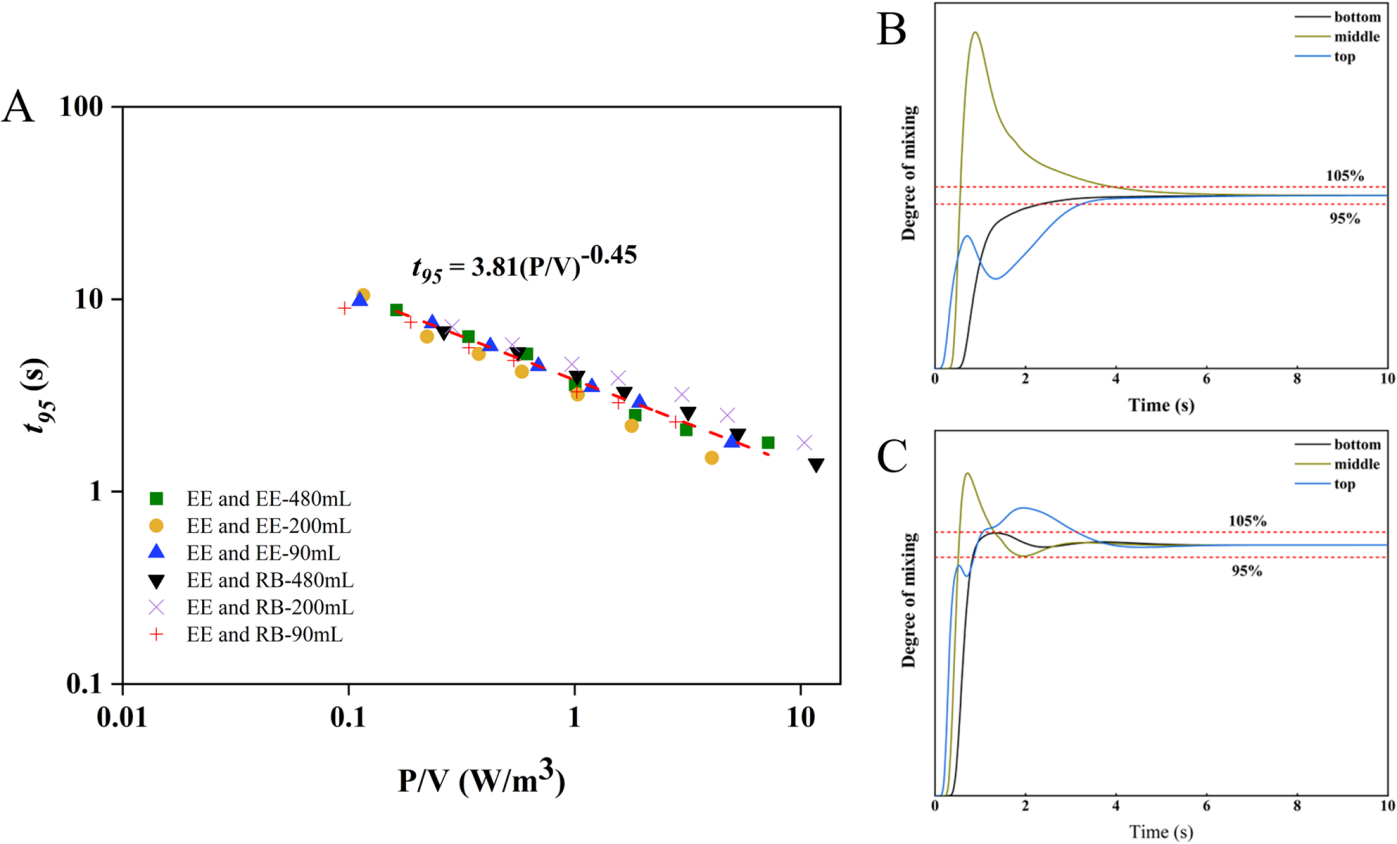
Mixing characteristics of single-use bioreactors under combination of EE-EE and EE-RB impellers under different operating conditions (**A** is $$ {t}_{95}$$ at different power per unit volume; **B** is the mixing curve at different level heights in the SUB with the impeller combination EE-EE; **C** is the mixing curve at different level heights in the SUB with the impeller combination EE-RB, Note: In panel **A**, both the X-axis and Y-axis are presented on a Log_10_ scale). EE-EE: double Elephant Ear impellers; EE-RB: Elephant Ear and Ribbon impellers

Efficient mixing in bioreactors ensures environmental homogeneity but requires balancing specific power input (P/V) with shear stress (TED and SSR) to prevent cell damage. In this study, the 95% mixing time (t_95_) was highly consistent across conditions (Fig. 11). While axial mixing was uniform at the bench scale, mitigating concentration gradients remains a critical consideration for ton-scale scale-up to prevent localized metabolic shifts. Following Vasconcelos et al. (2000), we correlated t_95_ and P/V as: $$ {t}_{95}=3.81{(P/V)}^{-0.45}$$. The scaling exponent − 0.45 deviates from the classical − 0.33 derived from Kolmogorov’s isotropic turbulence theory for fully turbulent flows (Re > 10^4^). This deviation is physically justified, as our bioreactor deliberately operates in the transitional flow regime to strictly minimize mechanical shear. Consequently, mixing relies predominantly on macro-convective bulk circulation driven by the dual-impeller configuration rather than on fully developed isotropic turbulence. Despite this low-shear operation, benchmarking analysis (Table 3) shows our system matches or exceeds analogous commercial bioreactors. Overall, the SUB achieves rapid mixing at exceptionally low power inputs, confirming its suitability for high-density mammalian cell culture.

### Cell perfusion culture

**Fig. 12:**
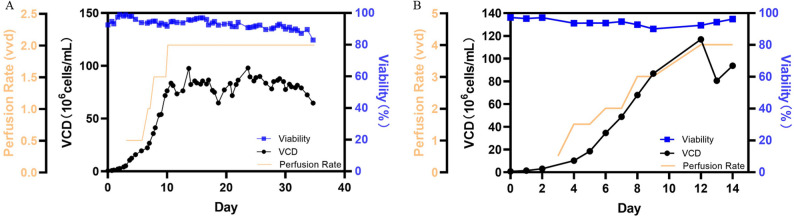
Results of cell culture (**A** is the culture result of CHO cells; **B** is the culture result of HEK293 cells)

The suitability of the Biogenstar bioreactor was rigorously evaluated through high-density perfusion cultures, serving as a validation of the engineering design. (1) For CHO cells, densities reached 8.32 × 10^7^ cells/mL on day 30 (Fig. 12A). Crucially, viability remained consistently above 90% throughout the production phase despite continuous agitation and ITF pumping. This phenotype provides direct biological validation of the CFD prediction that the average SSR (< 25 s^−1^) creates a hydrodynamically safe environment. (2) For HEK293 cells, the transition to perfusion mode drove a marked increase in growth rates, peaking at an ultra-high concentration of 1.17 × 10^8^ cells/mL(Fig. 12B). The achievement of such densities in the absence of oxygen limitation confirms that the bioreactor’s experimental $$ {k}_{L}a$$ (≈ 15 h^−1^) effectively meets biological oxygen demand. Additionally, the stable growth profile confirms that the rapid mixing time (1–10 s) successfully prevents nutrient gradients.

## Conclusions

To evaluate the suitability of the bioreactor system developed in this study for cell culture, we conducted comprehensive characterization from flow field simulation to high-density perfusion culture. CFD results predicted a “hydrodynamically safe” operating environment: featuring rapid mixing capability (1–10 s) and appropriate power input, with average SSR (< 25 s^−1^) and EDR values significantly below cell damage thresholds. This engineering prediction was directly validated in subsequent experiments: the reactor successfully supported ultra-high-density cultures of CHO and HEK293 cells (reaching 8.32 × 10^7^ and 1.17 × 10^8^ cells/mL respectively). Crucially, cell viability remained consistently above 90% throughout continuous agitation and ITF pumping during production, directly corroborating CFD predictions of a mild shear environment. Concurrently, high-density growth confirmed that *k*_*L*_*a* (reaching 15 h^−1^) adequately met the exceptionally high metabolic demands. In summary, this study demonstrates that the CFD-assisted design of the impeller and ITF system effectively resolves the trade-off between mixing/mass transfer efficiency and shear stress, proving Biogenstar highly suitable for scalable biopharmaceutical production. Furthermore, the quantitative hydrodynamic profiles established here provide a robust foundation for bioprocess scale-up, as maintaining constant P/V and SSR volumetric distributions across scales ensures a predictable physiological response and consistent performance in larger-volume bioreactors.

## Supplementary Information

Below is the link to the electronic supplementary material.

Supplementary Material 1

## Data Availability

Data are available by request to the corresponding author.

## Abbreviations

ATF: Alternating tangential flow filtration
CFD: Computational fluid dynamics
DO: Dissolved oxygen
EDR: Energy dissipation rate
EE: Elephant ear impeller
ITF: Impulse tangential-flow filtration
RB: Ribbon impeller
SSR: Shear strain rate
SUB: Single-use bioreactor
TED: Turbulence eddy dissipation
TFF: Tangential flow filtration
VCD: Viable cell density
VVD: Vessel volume per day
${{k}}_{{L}}{a}$: Volumetric oxygen transfer coefficient
P/V: Power input per unit volume
${{t}}_{95}$: Mixing time to reach 95% homogeneity
