# Supplementary material for: CFD-aided design and hydrodynamic characterization of a single-use perfusion bioreactor for high density cell culture
Source: Bioresour Bioprocess. 2026 Jun 3;13(1):84. doi: 10.1186/s40643-026-01077-6 (PMC13234075; doi:10.1186/s40643-026-01077-6)
Supplement: Supplementary file 1 — Supplementary Material 1 [file 40643_2026_1077_MOESM1_ESM.docx]

**Title**

**CFD-Aided design and hydrodynamic characterization of a single-use perfusion bioreactor for high density cell culture**

**S1. Mesh Independence Study**

To ensure the accuracy of the computational fluid dynamics (CFD) simulations and to eliminate the influence of grid resolution on the numerical results, a mesh independence study was conducted. Four different grid configurations were generated with total mesh numbers of 1.05, 1.6, 2.3, and 3.1 million elements, respectively. Global hydrodynamic parameters, including torque, average shear strain rate (ave SSR), average turbulent eddy dissipation (ave TED), and average velocity (ave velocity), were systematically monitored to evaluate grid convergence.

As shown in Figure S1 the monitored parameters exhibit noticeable variations when the mesh number increases from 1.05 million to 2.3 million. However, as the mesh density further increases from 2.3 million to 3.1 million, the macroscopic parameters plateau, showing negligible relative differences. For instance, the average velocity and average SSR remain almost constant between the 2.3 million and 3.1 million mesh schemes. Considering the critical balance between simulation accuracy and computational cost, the mesh configuration with 2.3 million elements was selected as the optimal grid baseline for all subsequent CFD simulations in this study.


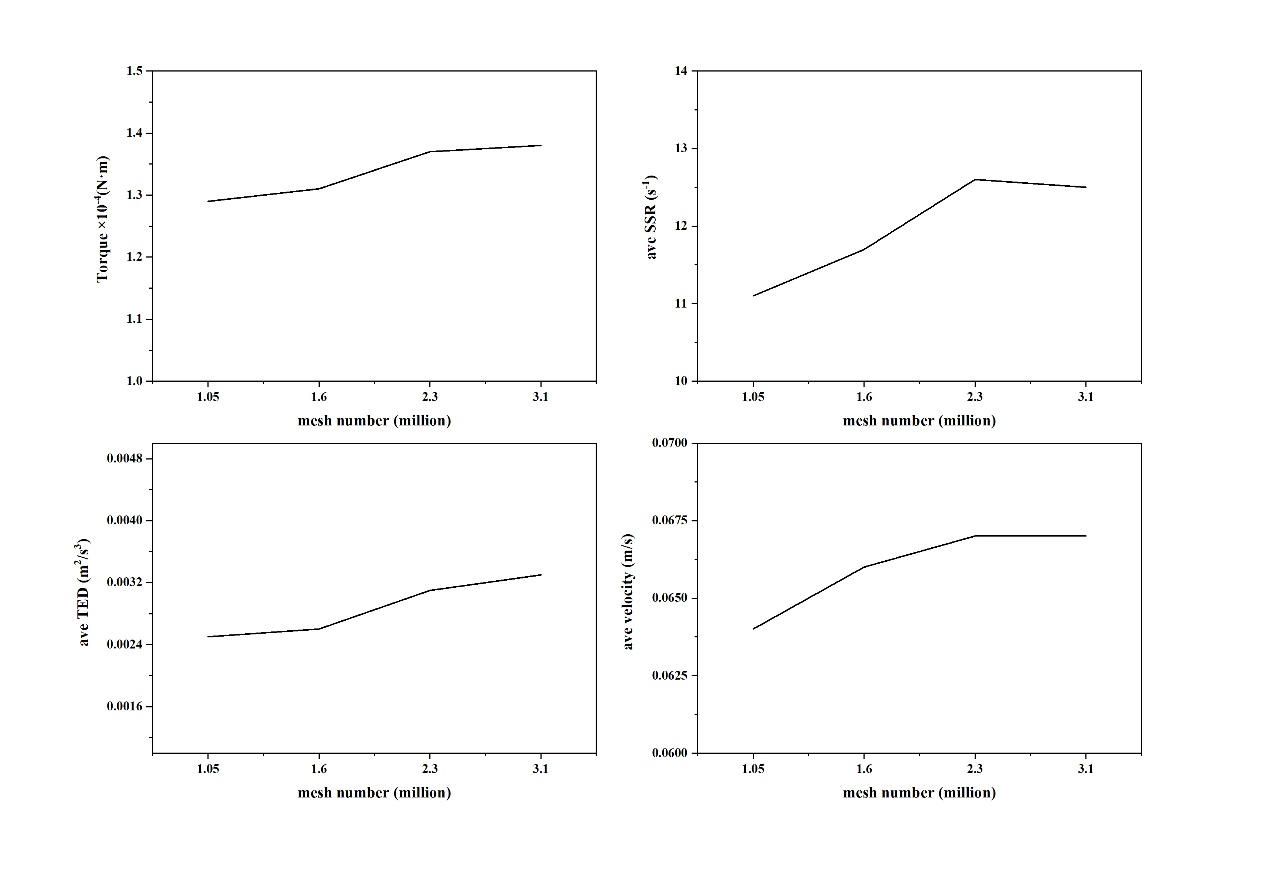


Figure. S1. Grid independence study for the bioreactor CFD model.
